# Inhibition of anti-tuberculosis T-lymphocyte function with tumour necrosis factor antagonists

**DOI:** 10.1186/ar1994

**Published:** 2006-07-19

**Authors:** Haïfa Hamdi, Xavier Mariette, Véronique Godot, Karin Weldingh, Abdul Monem Hamid, Maria-Victoria Prejean, Gabriel Baron, Marc Lemann, Xavier Puechal, Maxime Breban, Francis Berenbaum, Jean-Charles Delchier, René-Marc Flipo, Bertrand Dautzenberg, Dominique Salmon, Marc Humbert, Dominique Emilie

**Affiliations:** 1 INSERM UMR-S764, Service d'Hépato-Gastro-Entérologie and Service de Microbiologie-Immunologie Biologique, Hôpital Antoine Béclère, Assistance Publique – Hôpitaux de Paris (AP-HP), Institut Paris-Sud sur les Cytokines, Université Paris-Sud, INSERM U764, 32 rue des Carnets, 92140, Clamart, France; 2 Service de Rhumatologie, Hôpital Bicêtre, AP-HP, Université Paris-Sud, INSERM U802, 78 rue du Général Leclerc, 94275 Le Kremlin-Bicêtre, France; 3 Department of Infectious Disease and Immunology, Statens Serum Institut, Copenhagen S, 5 Artillerivej, 2300 Denmark; 4 Service de Pneumologie, Hôpital A. Béclère, AP-HP, Université Paris-Sud, 157 rue de la Porte-de-Trivaux, 92140 Clamart, France; 5 Département d'Epidémiologie, Biostatistique et Recherche Clinique, Groupe Hospitalier Bichat Claude-Bernard, AP-HP, Université Paris VII, INSERM U738, 46 rue Henri-Huchard, 75018 Paris, France; 6 Service de Gastro-entérologie, Hôpital St. Louis, AP-HP, 1 avenue Claude-vellefaux, 75475 Paris, France; 7 Service de Rhumatologie, Centre hospitalier du Mans, 194 avenue Rubillard, 72037 Le Mans, France; 8 Service de Rhumatologie, Hôpital A. Paré, AP-HP, 9 avenue Charles-de-Gaulle, 92100 Boulogne, France; 9 Service de Rhumatologie, Hôpital St. Antoine, AP-HP, 184 rue du Faubourg Saint-Antoine, 75012 Paris, France; 10 Service de Gastro-entérologie, Hôpital H. Mondor, AP-HP, 51 rue du Maréchal de Lattre de Tassigny, 94400 Créteil, France; 11 Service de Rhumatologie, Hôpital C. Huriez, rue Michel Polonovski, 59037 Lille, France; 12 Service de Pneumologie, Hôpital Pitié-Salpétrière, AP-HP, 83 boulevard de l'Hôpital, 75013 Paris, France; 13 Service de Médecine interne et maladies infectieuses, Hôpital Cochin, 27 rue du Faubourg Saint Jacques, 75014 Paris, France; 14 Service de Microbiologie – Immunologie Biologique, Hôpital A. Béclère, AP-HP Université Paris-Sud,, 157 rue de la Porte-de-Trivaux, 92140 Clamart, France

## Abstract

Reactivation of latent *Mycobacterium tuberculosis *(*Mtb*) infection is a major complication of anti-tumour necrosis factor (TNF)-α treatment, but its mechanism is not fully understood. We evaluated the effect of the TNF antagonists infliximab (Ifx), adalimumab (Ada) and etanercept (Eta) on anti-mycobacterial immune responses in two conditions: with *ex vivo *studies from patients treated with TNF antagonists and with the *in vitro *addition of TNF antagonists to cells stimulated with mycobacterial antigens. In both cases, we analysed the response of CD4^+ ^T lymphocytes to purified protein derivative (PPD) and to culture filtrate protein (CFP)-10, an antigen restricted to *Mtb*. The tests performed were lymphoproliferation and immediate production of interferon (IFN)-γ. In the 68 patients with inflammatory diseases (rheumatoid arthritis, spondylarthropathy or Crohn's disease), including 31 patients with a previous or latent tuberculosis (TB), 14 weeks of anti-TNF-α treatment had no effect on the proliferation of CD4^+ ^T lymphocytes. In contrast, the number of IFN-γ-releasing CD4^+ ^T lymphocytes decreased for PPD (*p *< 0.005) and CFP-10 (*p *< 0.01) in patients with previous TB and for PPD (*p *< 0.05) in other patients (all vaccinated with Bacille Calmette-Guérin). Treatments with Ifx and with Eta affected IFN-γ release to a similar extent. *In vitro *addition of TNF antagonists to CD4^+ ^T lymphocytes stimulated with mycobacterial antigens inhibited their proliferation and their expression of membrane-bound TNF (mTNF). These effects occurred late in cultures, suggesting a direct effect of TNF antagonists on activated mTNF^+ ^CD4^+ ^T lymphocytes, and Ifx and Ada were more efficient than Eta. Therefore, TNF antagonists have a dual action on anti-mycobacterial CD4^+ ^T lymphocytes. Administered *in vivo*, they decrease the frequency of the subpopulation of memory CD4^+ ^T lymphocytes rapidly releasing IFN-γ upon challenge with mycobacterial antigens. Added *in vitro*, they inhibit the activation of CD4^+ ^T lymphocytes by mycobacterial antigens. Such a dual effect may explain the increased incidence of TB in patients treated with TNF antagonists as well as possible differences between TNF antagonists for the incidence and the clinical presentation of TB reactivation.

## Introduction

Tumour necrosis factor (TNF) antagonists such as the anti-TNF monoclonal antibodies (mAbs) infliximab (Ifx) and adalimumab (Ada) and the soluble TNF receptor etanercept (Eta) are efficacious in several immune-mediated inflammatory diseases (IMIDs), including rheumatoid arthritis (RA), spondylarthropathies (SA), Crohn's disease (CD), psoriasis arthritis, and juvenile arthritis [1-8]. However, they are also associated with an increased incidence of infections, especially infection with *Mycobacterium tuberculosis *(*Mtb*). Tuberculosis (TB) in patients treated with TNF antagonists is characterised by a high frequency of extra-pulmonary and disseminated lesions and with few granulomas in involved organs. Because most cases of TB develop soon after treatment initiation, they correspond to a reactivation of a latent TB infection [9-11].

All three TNF antagonists have been associated with increased incidence of TB. However, this incidence seems to be lower for Eta than for Ifx [12,13], and the median delay between treatment initiation and occurrence of TB was shorter with Ifx [11]. Membrane-anchored TNF (mTNF) is expressed by activated macrophages and T lymphocytes [14,15]. Although Ifx and Eta both neutralise soluble TNF, Ifx binds more efficiently to mTNF than does Eta. Thus, Ifx but not Eta induces apoptosis of activated monocytes and lamina propria T lymphocytes from patients with CD [15,16]. The mechanism by which TNF antagonists reactivate latent TB is not fully understood. In animal models, TNF plays a central role in the containment of mycobacterial infections, and T cell-derived soluble TNF as well as mTNF are essential in protecting against *Mtb *infection [17-22].

Detection of latent TB is crucial before starting treatment with TNF antagonists because it requires a preventive treatment for TB reactivation before TNF antagonist administration [23-25]. However, this detection is difficult, especially in individuals vaccinated with the Bacille de Calmette Guérin (BCG). Diagnosis of latent TB may benefit from new *in vitro *assays testing the immune response against proteins such as culture filtrate protein (CFP)-10 and early secreted antigenic target (ESAT)-6, which are encoded in the genome of *Mtb *and of a few other mycobacterial species (*Mycobacterium kansasii*, *Mycobacterium szulgai*, and *Mycobacterium marinum*) but not in that of BCG and other mycobacteria. Presence of an immune response against CFP-10 and ESAT-6 is a relatively specific indicator of *Mtb *infection and has allowed for precise diagnosis of active as well as latent TB in several studies of BCG-vaccinated individuals [26-32].

In the present work, we analysed the effect of TNF antagonists on the immune response against mycobacterial antigens, either CFP-10 or purified protein derivative (PPD), which contains antigens shared by all mycobacterial species, including BCG. This effect was studied in two different conditions. In patients with an active form of RA, SA, or CD, the impact of treatment with TNF antagonists on circulating T lymphocytes was evaluated by analyzing *ex vivo *their proliferation and their rapid release of interferon (IFN)-γ in response to mycobacterial antigens. We also determined whether TNF antagonists added *in vitro *to blood cells alter their activation by mycobacterial antigens.

## Materials and methods

### Characteristics of patients

Patients were consecutively enrolled in the study between April 2003 and May 2005. They were divided into four groups, depending on previous or latent TB and IMID. Previous TB was defined as a previous known history of TB with adequate treatment. Latent TB was defined according to French recommendations [23]: a previous TB with no adequate treatment, a wheal larger than 10 mm in diameter or a blister in response to a tuberculin skin test (TST) performed more than 10 years after the last BCG vaccination, or radiographic evidence of residual nodular tuberculous lesions larger than 1 cm^3 ^in size. Adequate treatment was a treatment initiated after 1970 and lasting at least 6 months, including at least 2 months with the rifampin-pyrazinamide combination [23]. Group I patients (*n *= 37) had RA, SA, or CD and no previous or latent TB. Group II patients (*n *= 31) had RA, SA, or CD and previous or latent TB. Group III patients (*n *= 21) had no RA, SA, or CD but any of the following lung diseases: emphysema (*n *= 1), uninfected chronic obstructive pulmonary disease (*n *= 6), asthma (*n *= 1), primary arterial hypertension (*n *= 11), pulmonary embolism (*n *= 1), or infectious pneumonitis (*n *= 1) with complete recovery. They had no previous or latent TB. Group IV patients (*n *= 24) had no IMID but previous TB. Twenty had been treated for TB for a median 32 (12–52 interquartile range) years before inclusion. Because BCG vaccination in infancy was mandatory in France until 2004, all patients in this study were expected to be vaccinated with BCG. A specific inquiry revealed no patients without BCG vaccination.

Patients from groups I and II were naive of TNF antagonist treatment, and all required treatment with Ifx (RA, 3 mg/kg at week 0, 2, 6 and then every 8 weeks; SA, 5 mg/kg with the same schedule; CD, 5 mg/kg at week 0, 2, and 6), Ada (RA, 40 mg every other week), or Eta (RA or SA, 25 mg twice a week). Patients from groups I and II were tested twice, at inclusion and 14 weeks after initiation of anti-TNF treatment. Two patients from group II had been previously treated for TB, 6 and 11 years before inclusion, respectively. In the other group II patients, anti-TB treatment was initiated at least 3 weeks before administration of the TNF antagonist and given for a total of at least 8 weeks, according to French recommendations [23]. Anti-tuberculous treatment consisted of isoniazid + rifampin for 3 months, isoniazid alone for 9 months, or isoniazid + pyrazinamide for 3 months. Patients gave informed consent, and this study was reviewed and approved by the ethics committee. The main characteristics of patients are summarised in Table 1.

### Reagents

Tuberculin (PPD) was from Statens Serum Institut (Copenhagen S, Denmark), cytomegalovirus (CMV) was from Cambrex Bio Science (Emerailville, France), and *Tetanus toxoid *(TT) and *Candida *(Cd) antigens were from Sanofi Diagnostics Pasteur (Aulnay-sous-Bois, France). *Toxoplasma gondii *(Toxo) was prepared from rough extract of tachyzoites (Francis Derouin, Hôpital Saint Louis, Paris, France). Phytohemagglutinin was from Murex Diagnostics (Paris).

Recombinant CFP-10 was cloned as histidine-tagged products as previously described [33,34] and purified with the use of a Talon resin (Clontech, Broendbry, Denmark) in 8 M urea followed by fractionation on a Hitrap Q HP column (GE Healthcare, Little Chalfont, Buckinghamshire, UK, formerly Amersham Biosciences) in 3 M urea. The fractions were analysed by use of silver-stained SDS-PAGE and western blotting with an anti-histidine antibody (Clontech) and a polyclonal anti-*Escherichia coli *antibody (Dako, Glostrup, Denmark) to detect contaminants. Fractions more than 99% pure were pooled and dialysed with 25 mM Hepes (pH 8.5). PPD and CFP-10 were used at 1 μg/ml and 0.5 μg/ml, respectively. For *in vitro *assays with Ifx, Ada, or Eta, human immunoglobulin G1-Kappa purified from myeloma serum (Serotec, Sergy Saint-Christophe, France) was used as a control.

### *Ex vivo *and *in vitro *assays

Thymidine incorporation (studied at day 5 of culture) and (PKH)-26 dilution assays (studied at day 7 of culture) were performed as described [35,36]. The PKH-26 assay detects CD4^+ ^T lymphocyte proliferation at the single-cell level. Briefly, patients' peripheral blood mononuclear cells (PBMCs) were labeled with PKH-26, cultured in the presence of the antigen, and tested by flow cytometry for PKH-26 labeling and for the expression of CD3 and CD4. Each round of CD4^+ ^T-cell proliferation during culture leads to a half-decrease of PKH-26 labeling intensity. IFN release was studied after 18 hours of culture of 2 × 10^5 ^cells per well by means of an enzyme-linked immunosorbent spot (ELISPOT) assay (Diaclone, Besançon, France). CD4^+ ^T lymphocyte depletion from PBMCs was performed using Dynabeads^® ^CD4 coated beads with anti-CD4 mAb (Dynal Biotech, Compiègne, France).

To assess the *in vitro *effects of TNF antagonists, we determined proliferation using thymidine incorporation assays. These experiments were performed from the week-0 sample and when allowed by PBMC recovery, and response to mycobacterial antigens was tested as a priority to other antigens. Because the therapeutic range of residual serum concentration is between 1 and 10 μg/ml for the three drugs [2,37,38], we used 10 μg/ml of Eta, Ada, and Ifx unless specified. Median effective concentration (EC_50_) values were determined using the WinNonlin Professional software (v3.1; Pharsight Corporation, Mountain View, CA, USA). Anti-interleukin (IL)-12 blocking antibody and its isotype control were used at 10 μg/ml (R&D Systems, Lille, France). Cultures were performed in the presence of 10% heat-decomplemented human AB-serum. We verified that no complement activity remained after heat inactivation.

Analysis of mTNF expression on T cells was performed by flow cytometry with anti-CD3-phycoerythrin, anti-CD4-phycoerythrin-cyanin-5.1 (Beckman Coulter, Villepinte, France), and anti-human extracellular TNF-fluorescein-isothiocyanate (clone 6,401) (R&D Systems) mAbs.

### Statistical analysis

Results were analysed with use of the non-parametric Mann-Whitney *U *test for descriptive evaluations between patients with or without TB. The Wilcoxon test was used for comparisons of paired values. Bonferroni corrections were performed for multiple comparisons. A *p *< 0.05 was considered as significant.

## Results

### Immune responses to mycobacterial antigens in patients with IMID before treatment with TNF antagonists

**Figure 1:**
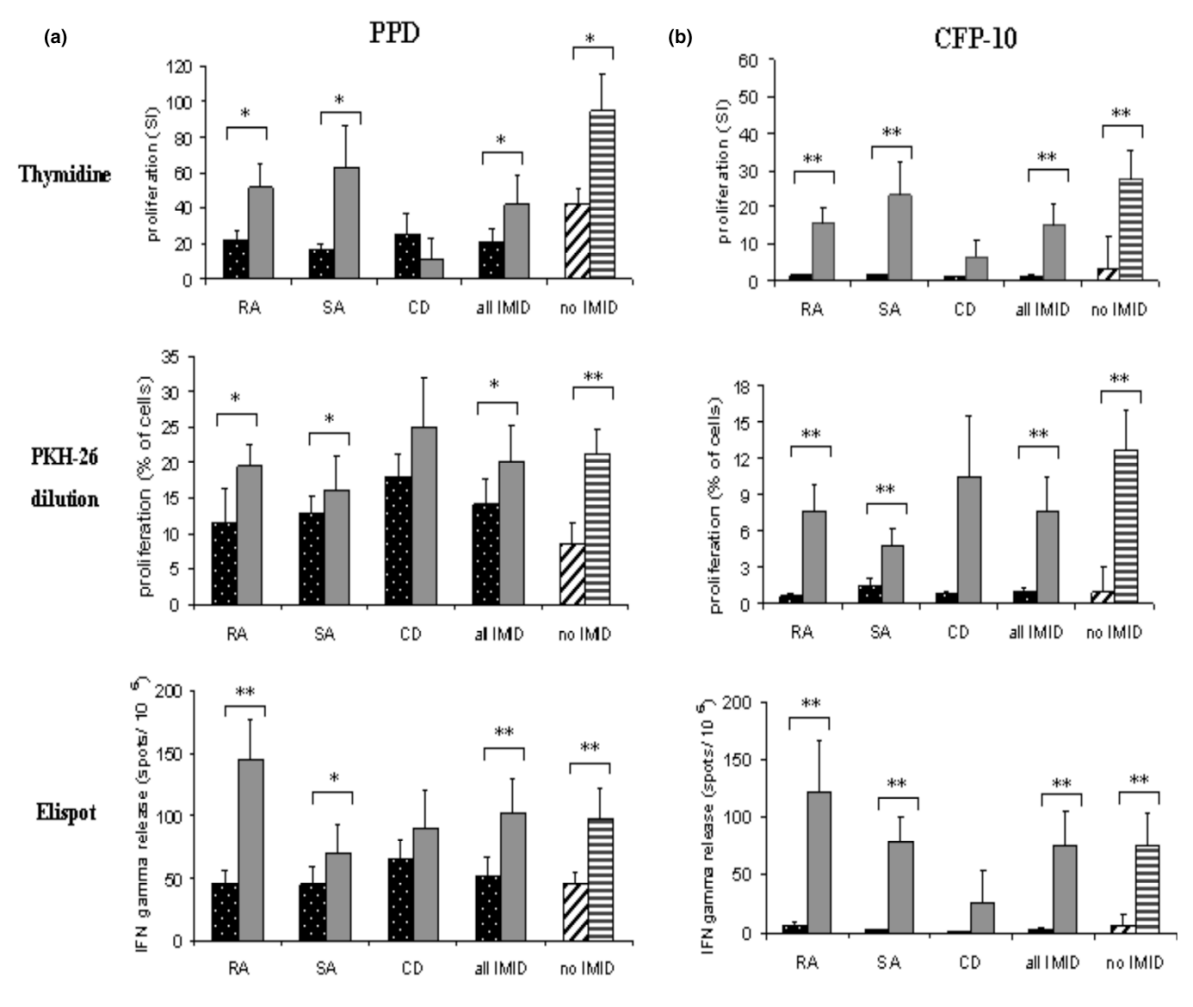
Responses to mycobacterial antigens in patients with or without an immune-mediated inflammatory disease (IMID). The response to purified protein derivative (PPD) **(a) **and to culture filtrate protein (CFP)-10 **(b) **was analysed using the thymidine incorporation, (PKH)-26 dilution, and enzyme-linked immunosorbent spot (ELISPOT) assays in patients with (groups I, dotted boxes and II, gray shaded boxes) or without (groups III, diagonally lined boxes and IV, horizontally lined boxes) an IMID, and with previous or latent tuberculosis (groups II and IV) or without (groups I and III). Group I included 13, 12, and 12 patients with rheumatoid arthritis (RA), sponlylarthropathy (SA), and Crohn's disease (CD), respectively. Group II included 16, 13, and 2 patients with RA, SA, and CD, respectively. Twenty-one and 24 patients were studied in groups III and IV, respectively. The thymidine incorporation, (PKH)-26 dilution, and enzyme-linked immunosorbent spot (ELISPOT) assays were performed, with results expressed (mean ± standard error of the mean) as stimulation index (SI), fraction of proliferating cells (percentage), and number of interferon (IFN)-γ-producing cells per 10^6 ^cells. Comparisons for each assay were between groups I and II and between groups III and IV (*p < 0.05 and **p < 0.005, Mann-Whitney *U *test).

The response to PPD and to the TB-specific antigen CFP-10 was compared between patients with or without an IMID. Three different assays were performed. Two of them tested lymphocyte proliferation, analyzing either PBMC (thymidine incorporation assay) or CD4^+ ^T lymphocytes (PKH-26 dilution assay). The third assay evaluated the number of IFN-γ-releasing cells (ELISPOT assay). The response against PPD was stronger in patients with a previous TB (*p *< 0.05 for comparisons between groups I and II, and between groups III and IV) (Figure 1a). However, when patients with and without an IMID were compared (regardless of the assay used), the intensity of the PPD-induced response was in the same range (group I versus II and group III versus IV, respectively, *p *> 0.05 for all comparisons). A response to CFP-10 was observed only in patients with a previous TB, regardless of the presence of an IMID (*p *< 0.005) (Figure 1b).

When considering RA and SA independently, similar conclusions were reached: responses induced by PPD and CFP-10 were in the same range as those of controls, for the three assays (Figure 1a,b). The limited number of patients with CD and previous TB (*n *= 2) precluded definitive conclusion regarding this group. Thus, neither the underlying IMID nor its treatment affected the *ex vivo *intensity of lymphocyte responses to mycobacterial antigens.

**Figure 2:**
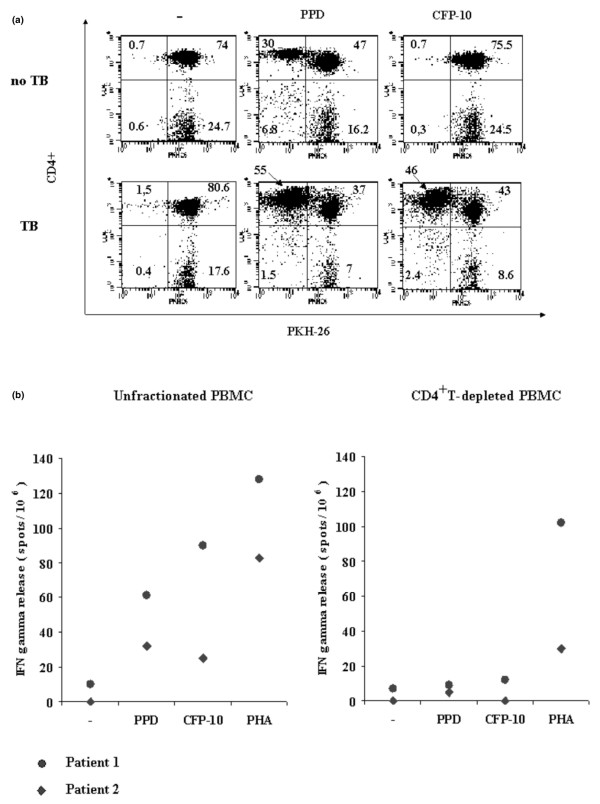
CD4^+ ^T lymphocytes are the main cells responding to *Mycobacterium tuberculosis *antigens. (a) Proliferative responses ([PKH]-26 assay) to purified protein derivative (PPD) and to culture filtrate protein (CFP)-10 are shown for two individuals, one without (no tuberculosis [TB]) and the other with a previous or latent TB. Dot plots are gated on CD3^+ ^cells and analyse the fluorescence intensity for both CD4 and PKH-26. Proliferating cells are PKH-26^low^. The percentage of CD3^+ ^cells in each quadrant is shown. (b) The release of interferon (IFN)-γ in response to PPD, CFP-10, or phytohemagglutinin (PHA) was tested using the enzyme-linked immunosorbent spot assay, evaluating the response of either unfractionated peripheral blood mononuclear cells (PBMCs) (left) or PBMCs depleted of CD4^+ ^T lymphocytes (right). Results are from two individuals with previous or latent TB and are expressed as the number of IFN-γ-releasing cells per million cells.

**Figure 3:**
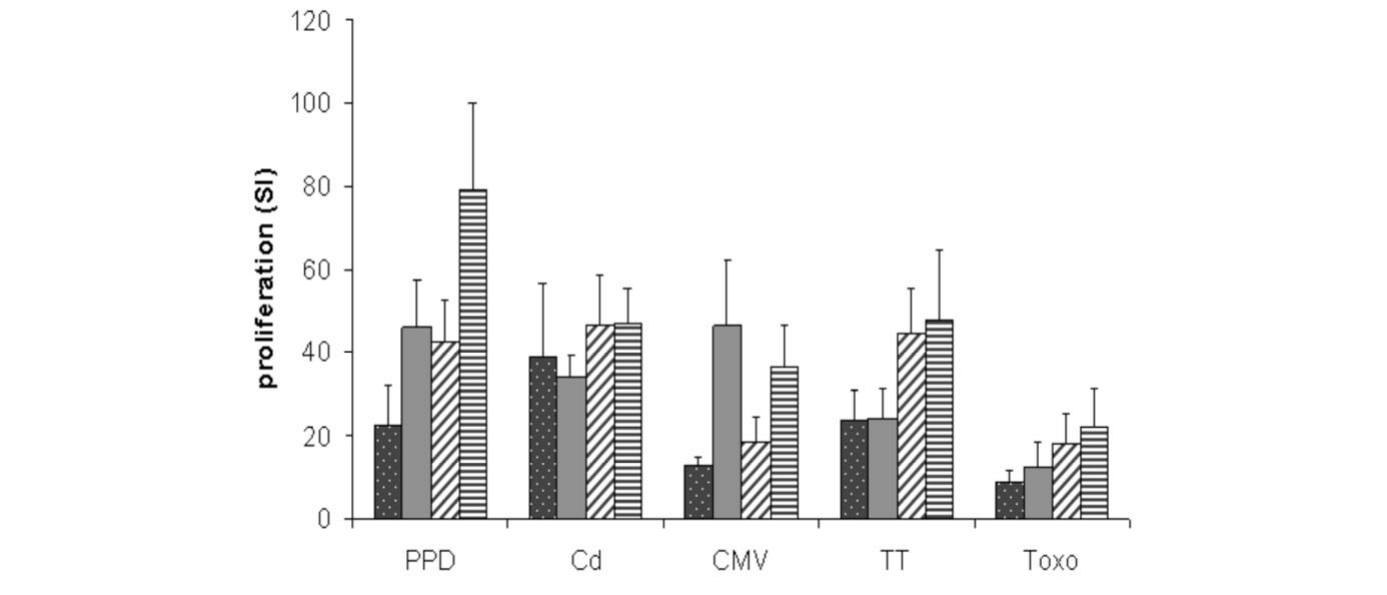
Proliferative responses to recall antigens in patients with immune-mediated inflammatory disease. The proliferative response to purified protein derivative (PPD), *Candida *(Cd), cytomegalovirus (CMV), *Tetanus toxoid *(TT), and *Toxoplasma gondii *(Toxo) was evaluated using the thymidine incorporation assay. Patients were from group I (dotted boxes) (*n *= 28) and from group II (gray shaded boxes) (*n *= 26), before TNF (tumour necrosis factor) antagonist administration, and from group III (diagonally lined boxes) (*n *= 20) and from group IV (horizontally lined boxes) (*n *= 14). Results are expressed as stimulation indices (SIs) (mean ± standard error of the mean). *P *> 0.05 for all comparisons between groups I and III and between groups II and IV, Mann-Whitney *U *test.

Proliferation in response to PPD and to CFP-10 was mostly restricted to CD4^+ ^T lymphocytes, with only a few CD4^- ^T lymphocytes proliferating when stimulated by these antigens (Figure 2a). CD4-depletion experiments also showed that CD4^+ ^cells were the major IFN-γ-releasing cells in the ELISPOT assay (Figure 2b). Analysis of proliferative responses to mycobacterial-unrelated antigens (Cd, CMV, TT, and Toxo) also revealed no significant difference between patients with or without an IMID (Figure 3).

### Evolution of anti-mycobacterial immune responses in patients treated with TNF antagonists

**Figure 4:**
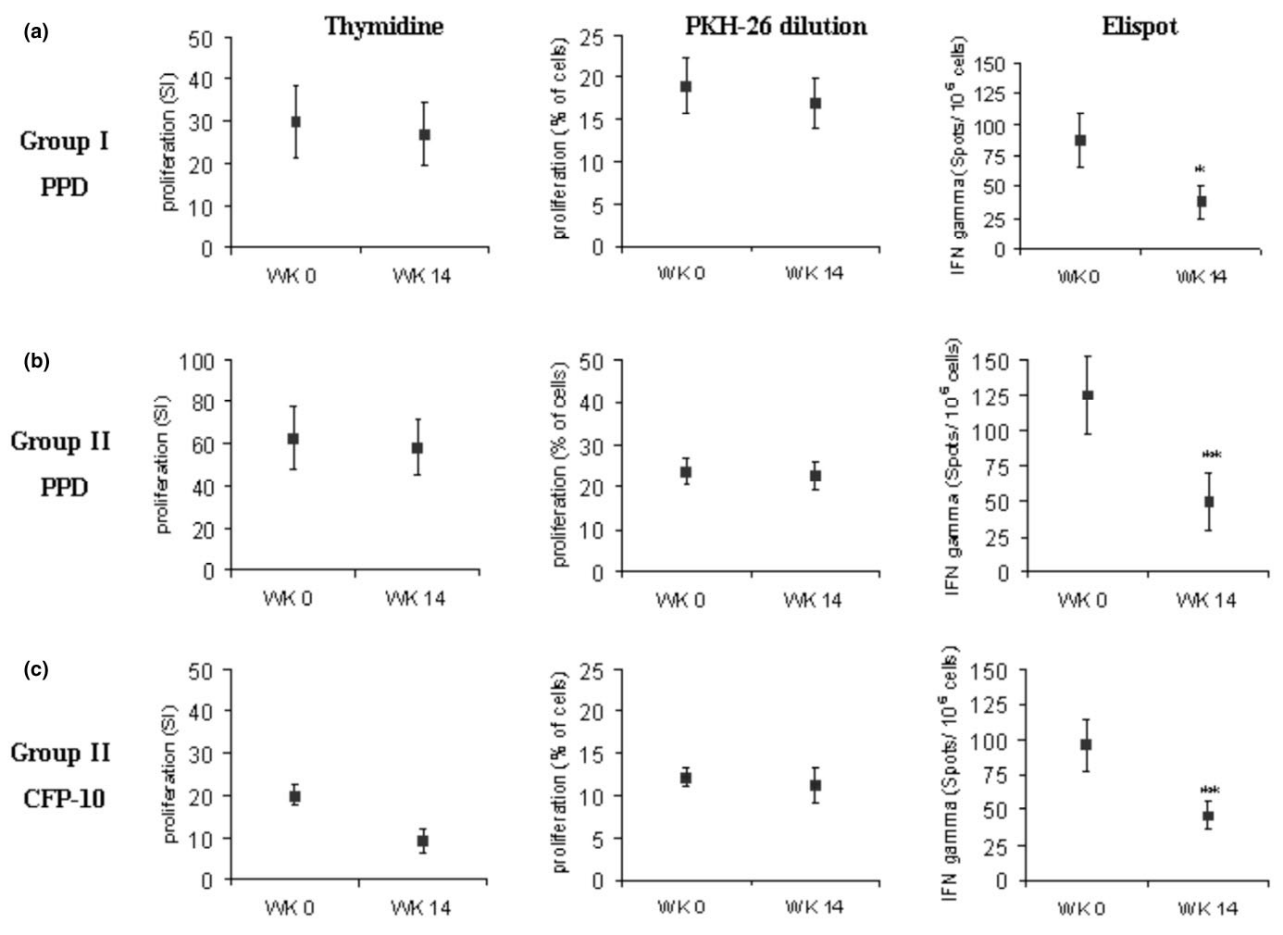
Effect of tumour necrosis factor (TNF) antagonist treatment on anti-mycobacterial responses. The effect of 14-week treatment with a TNF antagonist on the immune response against purified protein derivative (PPD) was determined in patients without a previous or latent tuberculosis group I **(a)**, and in patients with a previous or latent tuberculosis group II **(b)**. The effect of 14-week treatment with a TNF antagonist on the immune response against culture filtrate protein (CFP)-10 was determined in patients with a previous or latent tuberculosis group II **(c)**. The thymidine incorporation, (PKH)-26 dilution, and enzyme-linked immunosorbent spot (ELISPOT) assays were performed, with results expressed (mean ± standard error of the mean) as stimulation indice (SI), fraction of proliferating cells (percentage), and number of interferon (IFN)-γ-producing cells per 10^6 ^cells. *n *= 31 in (a) and *n *= 25 in (b) and (c). *p < 0.01, **p < 0.005 for comparisons between week 14 and week 0 (Wilcoxon test).

The intensity of the immune response against PPD and CFP-10 was determined in patients from groups I and II at inclusion and 14 weeks after initiation of TNF antagonist treatment. Treatment had no effect on the intensity of the proliferative response, regardless of the test used or of the mycobacterial antigen tested (*p *> 0.05 for all comparisons). In contrast, the number of IFN-γ-releasing cells in response to PPD (for groups I and II) and to CFP-10 (for group II) significantly decreased with treatment (Figure 4). This decrease was independent of an associated anti-TB treatment. The anti-PPD response indeed decreased in group I patients, who required no anti-TB treatment before administration of TNF antagonists. The anti-PPD and anti-CFP-10 responses also decreased in the two patients from group II previously treated for TB and thus needing no additional anti-TB treatment. In these two patients, the number of IFN-γ-releasing cells decreased by an average 89% and 72% for PPD and CFP-10, respectively. TNF antagonists decreased the number of IFN-γ-releasing cells to the same extent in patients with RA or SA (data not shown). These findings indicate that *in vivo *administration of TNF antagonists decreases the number of anti-TB CD4^+ ^T lymphocytes immediately releasing IFN-γ in response to mycobacterial antigens, whereas this treatment does not affect proliferative responses to the same antigens.

**Figure 5:**
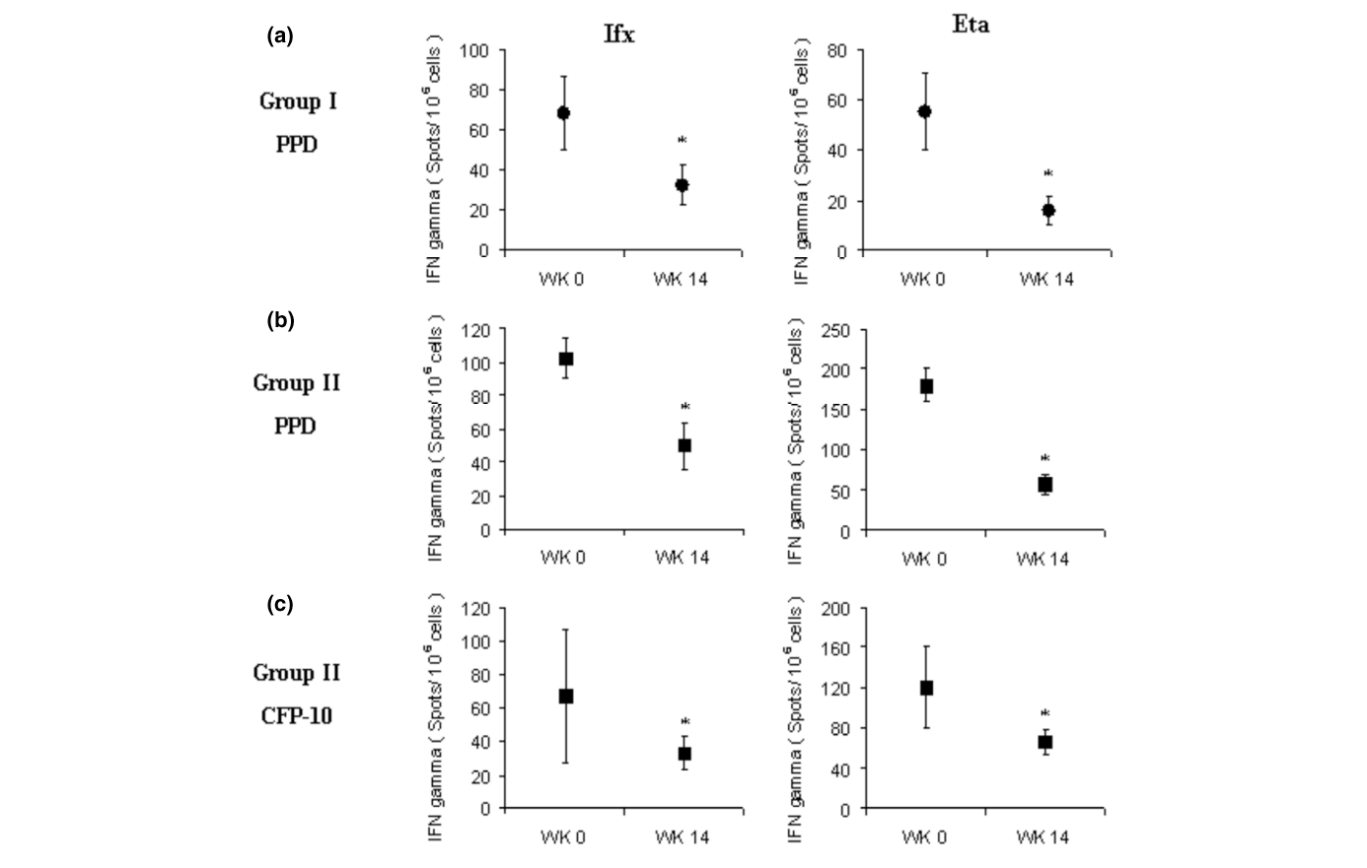
Effect of infliximab (Ifx) and etanercept (Eta) treatment on anti-mycobacterial responses. The effect of 14-week treatment with Ifx or with Eta on interferon (IFN)-γ release induced by purified protein derivative (PPD) in patients from group I, **(a) **or in patients from group II **(b)**. The effect of 14-week treatment with Ifx or with Eta on interferon (IFN)-γ release induced by culture filtrate protein (CFP)-10 was determined in patients from group II **(c)**. Results are expressed (mean ± standard error of the mean) as number of interferon (IFN)-γ-producing cells per 10^6 ^cells. *n *= 13 and 16 for Ifx and Eta respectively in (a). *n *= 11 for both Ifx and Eta in (b) and (c). *p < 0.01 for comparisons between week 0 and week 14 (Wilcoxon test).

We compared Ifx and Eta for their effect on IFN-γ release; because of their limited numbers, patients treated with Ada could not be tested for this. Treatments with Ifx or Eta decreased the number of IFN-γ-releasing cells in response to both PPD (in groups I and II patients) and CFP-10 (in group II patients) (Figure 5).

### *In vitro *effects of TNF antagonists on the activation of anti-mycobacterial T lymphocytes

**Figure 6:**
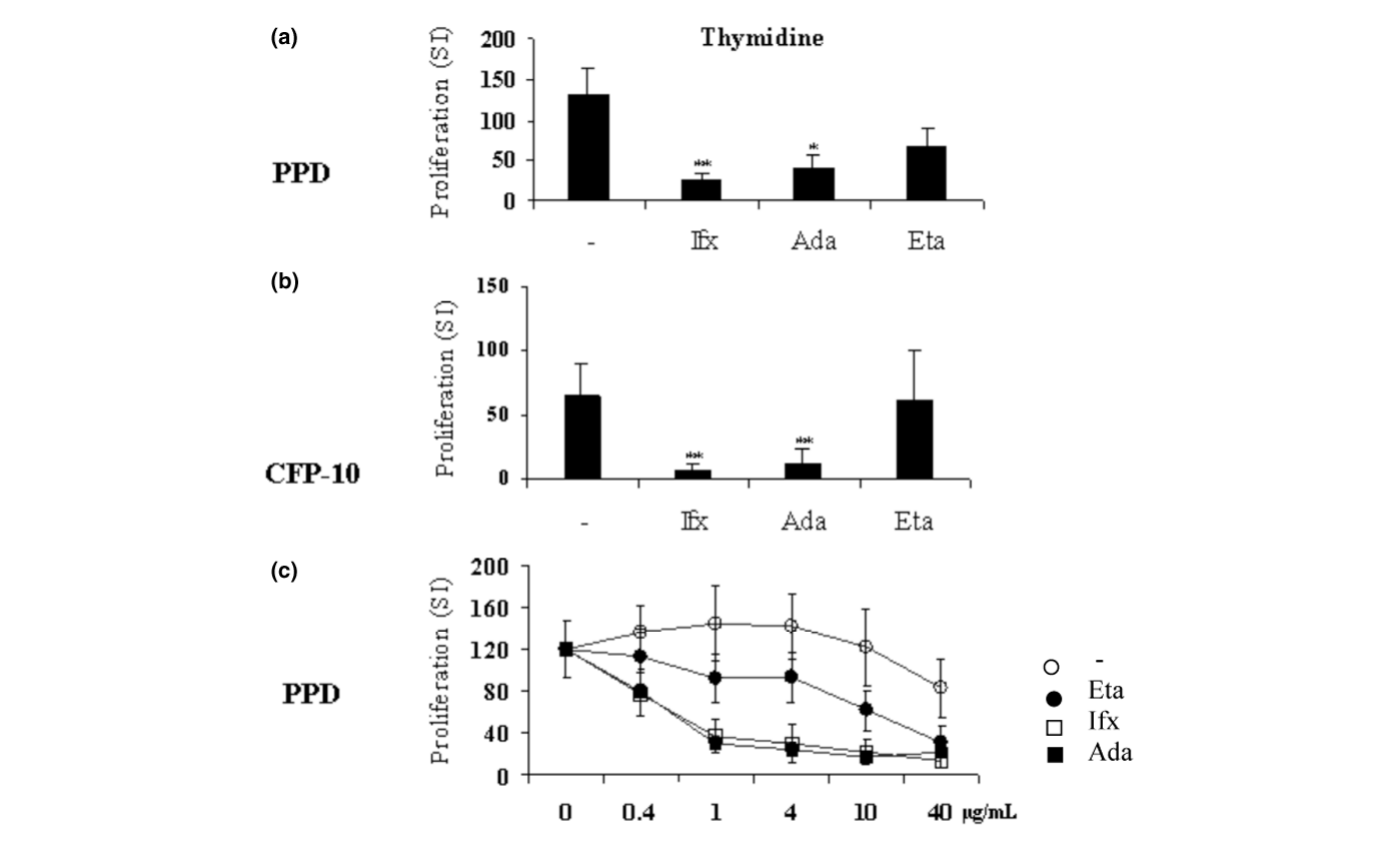
*In vitro *effect of tumour necrosis factor (TNF) antagonists on anti-mycobacterial proliferative response. In patients with previous or latent TB, lymphocyte proliferation was determined using the thymidine incorporation assay after 5 days of activation with purified protein derivative (PPD) in the presence of 10 μg/ml of a control antibody (-) or of infliximab (Ifx), adalimumab (Ada), or etanercept (Eta) **(a)**. In patients with previous or latent TB, lymphocyte proliferation was determined using the thymidine incorporation assay after 5 days of activation with culture filtrate protein (CFP)-10 in the presence of 10 μg/ml of a control antibody (-) or of infliximab (Ifx), adalimumab (Ada), or etanercept (Eta) **(b)**. Twelve patients were tested: four from group II, before TNF antagonist administration, and eight from group IV, with similar findings in both cases. **(c) **Graded concentrations of TNF antagonists were added to PPD-stimulated cells (*n *= 9). Results are expressed as stimulation indices SIs (mean ± standard error of the mean (SEM). *p < 0.05 for paired comparison between Ifx, Ada, or Eta and the control antibody (Wilcoxon test with Bonferroni corrections).

**Figure 7:**
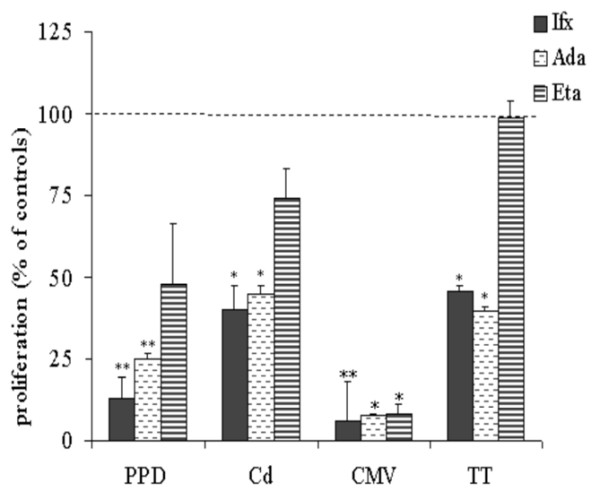
*In vitro *effect of tumour necrosis factor antagonists on proliferative responses to recall antigens. The effect of infliximab (Ifx), adalimumab (Ada), and etanercept (Eta) (10 μg/ml) on the proliferation of lymphocytes was determined using the thymidine incorporation assay after 5 days of activation with purified protein derivative (PPD), *Candida *(Cd), cytomegalovirus (CMV), or *Tetanus toxoid *(TT). Five patients (3 from group III and 2 from group IV) were tested. Results (mean ± standard error of the mean) are expressed as percentage of controls, corresponding to cells cultured with a control antibody. *p < 0.05 and **p < 0.01 as compared with controls (Wilcoxon test with Bonferroni corrections).

The above experiments analysed the impact of TNF antagonist treatment on the *in vivo *persistence of anti-mycobacterial CD4^+ ^T lymphocytes. Because no patients suffered from active TB, these lymphocytes were presumably resting memory T lymphocytes at the moment of blood collection. To analyse the effect of TNF antagonists on the activation of anti-mycobacterial CD4^+ ^T lymphocytes, we performed *in vitro *studies in which TNF antagonists were added to PBMC cultures stimulated with mycobacterial antigens. All patients tested had a previous or latent TB, and none of them received TNF antagonists when blood was collected. Ifx and Ada at 10 μg/ml inhibited PPD-induced proliferation, both in thymidine incorporation and in PKH-26 dilution assays. The responses in the presence of Eta (10 μg/ml) did not significantly differ from those of controls (Figure 6a,b). Similar findings were observed when testing CFP-10-induced proliferation (data not shown). However, increasing the dose of Eta above 10 μg/ml inhibited the anti-PPD response (Figure 6c). The EC_50 _(standard error of the mean) values for Ifx, Ada, and Eta were 10.7 (2.0) μg/ml, 7.1 (2.0) μg/ml, and 21.6 (9.0) μg/ml, respectively. Similar findings were observed for the anti-CFP-10 response (data not shown). Therefore, all TNF antagonists added *in vitro *inhibited the proliferative response of activated anti-mycobacterial CD4^+ ^T lymphocytes, but the concentration of Eta had to be two to three times higher than that of Ifx or Ada to obtain this effect. To address whether the effect of TNF antagonists (10 μg/ml each) on T-lymphocyte activation was restricted to the anti-PPD response, we tested their effect on the proliferative response against Cd, CMV, and TT. Ifx and Ada significantly inhibited the response to all antigens. Eta strongly inhibited the response to CMV, whereas it had no significant effect on the response against the other antigens (Figure 7).

### *In vitro *effects of TNF antagonists on mTNF expression by CD4^+ ^T lymphocytes

**Figure 8:**
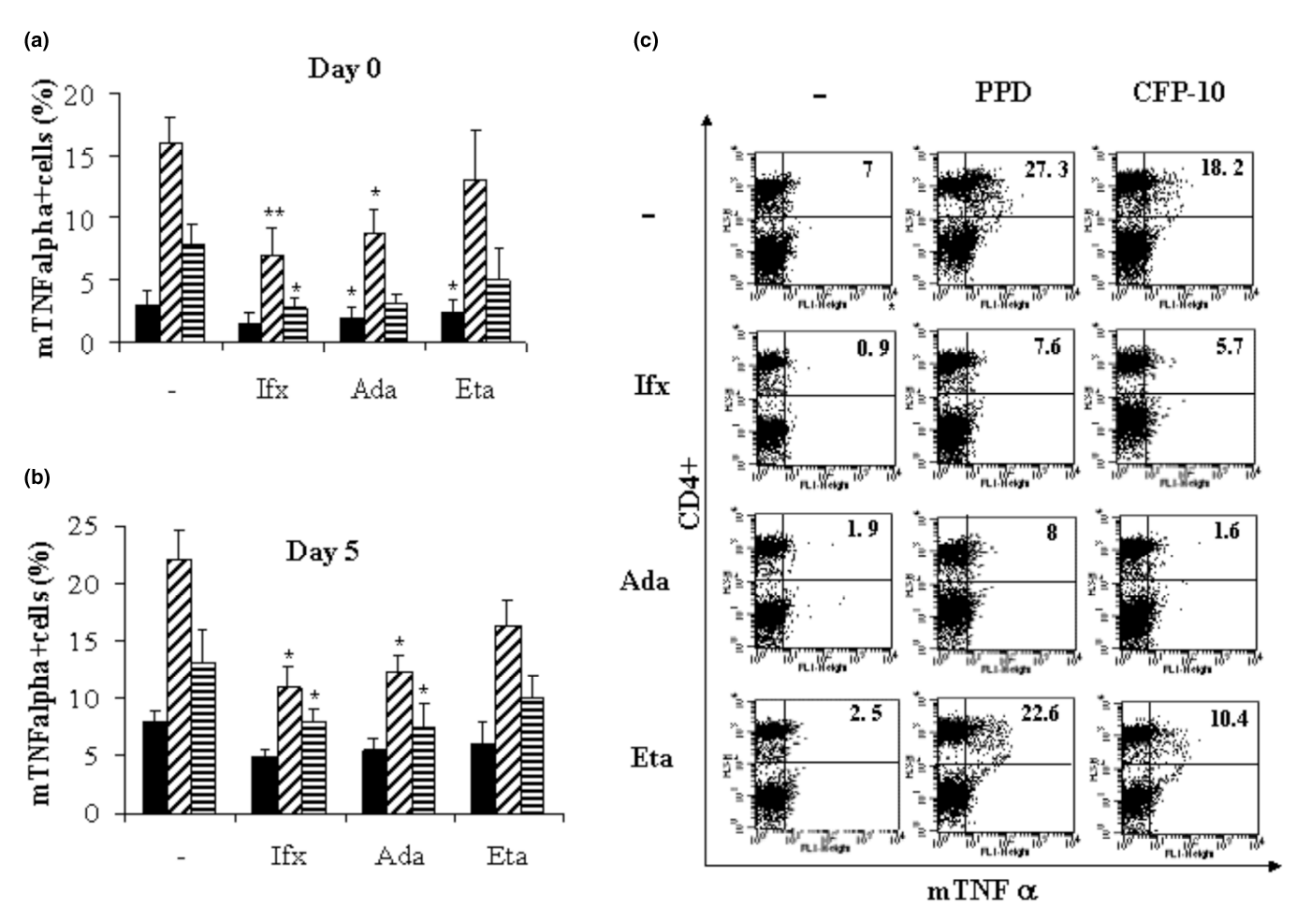
*In vitro *effect of tumour necrosis factor (TNF) antagonists on membrane-bound TNF (mTNF) expression. In patients previously infected with TB (either from group II or IV), peripheral blood mononuclear cells were cultured alone (black shaded boxes), with purified protein derivative (PPD) (diagonally lined boxes), or with culture filtrate protein (CFP)-10 (horizontally lined boxes), and with infliximab (Ifx), adalimumab (Ada), etanercept (Eta), or a control antibody (-) (10 μg/ml each). (a) The TNF antagonist was added at the initiation of the culture, and mTNF expression on CD4^+ ^T lymphocytes was determined on day 5 (*n *= 10). (b) The TNF antagonist was added on day 5 of culture, and mTNF expression on CD4^+ ^T lymphocytes was determined 24 hours later (*n *= 7). Results (mean ± standard error of the mean) are expressed as the fraction of mTNF^+ ^cells among CD4^+ ^T lymphocytes. Paired comparisons were performed. *p < 0.05 (Wilcoxon test with Bonferroni corrections) for comparisons between cultures with Ifx, Ada, or Eta and cultures with the control antibody. (c) Results from a typical patient in the same conditions as in (b).

To begin to determine the mechanism of action of TNF antagonists on T-lymphocyte activation, we quantified mTNF expression by CD4^+ ^T lymphocytes in cultures stimulated with mycobacterial antigens. PPD stimulation increased the fraction of mTNF-expressing CD4^+ ^T lymphocytes. CFP-10 stimulation also increased this expression, although to a lesser extent. The addition at the initiation of culture of either Ifx or Ada (10 μg/ml) decreased the fraction of mTNF-expressing CD4^+ ^T lymphocytes (Figure 8a). This effect persisted when Ifx/Ada addition was delayed up to day 5 of culture (that is, 24 hours before assessing mTNF expression on CD4^+ ^T lymphocytes) (Figure 8b,c). Eta had no effect on mTNF expression, regardless of the moment of its addition (Figure 8a–c).

**Figure 9:**
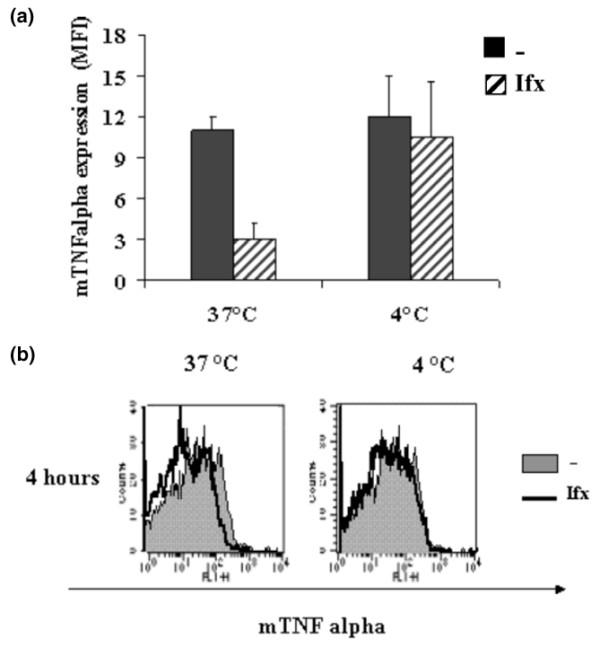
Expression of membrane-bound tumour necrosis factor (mTNF) after 4 hours of incubation with infliximab (Ifx). Cells stimulated for 6 days with purified protein derivative were incubated with Ifx (10 μg/ml) or a control monoclonal antibody at 4°C or at 37°C for 4 hours. Expression of mTNF on CD4^+ ^T lymphocytes was then determined. (a) Results are expressed as mean fluorescence intensity (MFI) ± standard error of the mean from three experiments. (b) One of these experiments is shown.

To analyse the mechanism involved in the downregulation of mTNF expression, we stimulated PBMCs with PPD for 5 days. Cells were stained with an anti-TNF mAb (clone 6401) before and after incubated with Ifx or a control antibody at either 4°C or 37°C for 4 hours. With Ifx, mTNF expression by CD4^+ ^T lymphocytes decreased at 37°C but not at 4°C (Figure 9). Results at 4°C showed an absence of competition between Ifx and the anti-TNF mAb (clone 6,401) used to stain the cells. Thus, the decreased expression of mTNF reported in Figure 8 cannot be explained by competition during labeling. In contrast, results at 37°C showed that Ifx induces a rapid disappearance of mTNF from CD4^+ ^T lymphocytes, involving an active process that could be either mTNF internalization or shedding.

### Delayed addition of TNF antagonists inhibits anti-mycobacterial proliferative responses

**Figure 10:**
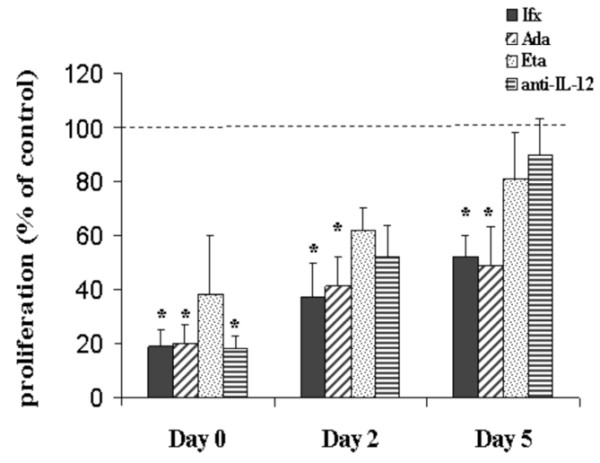
Delayed effect of tumour necrosis factor antagonists on lymphocyte proliferation. Infliximab (Ifx), adalimumab (Ada), etanercept (Eta), or an anti-interleukine (IL)-12 monoclonal antibody (mAb) was added either at the initiation of culture or on day 2 or 5 to peripheral blood mononuclear cells stimulated with purified protein derivative. Thymidine incorporation was determined on day 7. Results (mean ± standard error of the mean) are expressed as the proportion of controls (performed in the presence of a control antibody). *p < 0.05 and **p < 0.01. *n *= 10 for Ifx, Ada, and Eta, and *n *= 3 for the anti-IL-12 mAb.

The effect of TNF antagonists on mTNF expression suggested that they directly acted on activated T lymphocytes. To further support such a hypothesis, we determined whether delaying the addition of TNF antagonists up to day 5 of cultures still inhibited lymphocyte proliferation induced by PPD. Delaying the addition of Ifx or Ada (10 μg/ml) decreased the magnitude of inhibition, but the inhibition persisted. Eta (10 μg/ml) had no significant effect (Figure 10). Similar findings were observed for the response to CFP-10 (data not shown).

Addition of a neutralizing anti-IL-12 mAb at the initiation of cultures inhibited the proliferation induced by PPD or CFP-10, showing the contribution of IL-12 (a product of antigen-presenting cells) in anti-mycobacterial proliferative responses. This effect was lost when the anti-IL-12 mAb was added later, either on day 2 or on day 5 (Figure 10 and data not shown). IL-12 was thus not required during the last days of lymphocyte proliferation. Therefore, Ifx and Ada affected a late event in lymphoproliferative responses, independently of IL-12, and this is consistent with a direct effect of TNF antagonists on T lymphocytes activated by mycobacterial antigens.

## Discussion

In this work, we studied the impact of IMIDs and their classical treatment on anti-*Mtb *immune responses and we evaluated the effect of TNF antagonists on such responses, using the recently identified *Mtb*-specific antigens CFP-10 and ESAT-6 and newly developed immunological assays. Because results with CFP-10 and ESAT-6 were identical in all instances, only findings with CFP-10 are reported in the present work. We studied the impact of treatment with TNF antagonists on the *ex vivo *response of circulating anti-*Mtb *T lymphocytes and the effect of TNF antagonists added *in vitro *during activation of these cells by mycobacterial antigens.

Assays based on the quantification of IFN-γ-releasing cells allow diagnosis of active TB [31], recent primary infection [30,32], and latent TB [28]. Our findings extend these previous reports in several aspects. We show that, in addition to IFN-γ release, proliferative responses induced by *Mtb*-specific antigens are witnesses of a prior contact with *Mtb*. We also show that in patients with IMIDs and before initiation of treatment with TNF antagonists, *ex vivo *evaluation of anti-*Mtb *immune responses accurately reflects previous or latent TB because our biological findings correlate well with the current investigation of previous or latent TB. Additional studies are in progress to determine which combination of mycobacterial antigens and assays is optimal to diagnose latent TB and whether it compares favorably with the TST.

Our results also show that the intensity of anti-*Mtb *immune responses was preserved in patients with IMIDs as compared with patients without. This was observed regardless of the IMID considered, suggesting that neither an IMID nor its classical treatment significantly affects anti-mycobacterial CD4^+ ^T lymphocytes. Independently of TNF antagonist treatments, there are controversies concerning the impact of IMID on anti-mycobacterial immune responses. Berg et al. showed a decreased response to PPD in patients with RA [39]. These authors measured IFN-γ production by enzyme-linked immunosorbent assay 7 days after *in vitro *stimulation of PBMCs, an assay clearly different from those we used (proliferation and immediate IFN-γ release tested by ELISPOT). Likewise, a decreased intensity of TST in patients with RA has been noted [40], but this finding was not confirmed in two other recent studies [41,42].

TNF antagonists increase the incidence and the severity of TB. It was thus of interest to demonstrate that TNF antagonists act *in vivo *on anti-TB immune cells and to define the type of immune response targeted by these agents. Proliferation in response to mycobacterial antigens remained unaffected 14 weeks after initiation of treatment with TNF antagonists. This negative finding is significant because in patients with latent TB and receiving no anti-TB treatment, reactivation of TB peaks 12 weeks after initiation of Ifx treatment [11]. In contrast to the preservation of proliferative responses, immediate release of IFN-γ was affected by the administration of TNF antagonists. The number of lymphocytes releasing IFN-γ within 18 hours after challenge with mycobacterial antigens significantly decreased 14 weeks after initiation of treatment, as compared with pre-treatment values. In most patients with previous or latent TB (group II patients), an anti-TB treatment was associated with TNF antagonists, raising the hypothesis that anti-TB treatment rather than TNF antagonist treatment decreased the number of IFN-γ-releasing lymphocytes. In patients with active TB, anti-TB treatment is indeed associated with a rapid decline of the anti-TB immune response, as assessed by ELISPOT [28]. In this condition, the ELISPOT assay evaluates not only resting memory cells, but also T lymphocytes recently activated *in vivo *by TB antigens. Such activated lymphocytes presumably vanish while TB replication stops. In patients with latent TB, it is unknown whether the anti-TB treatment affects the number of IFN-γ-releasing cells. Although we cannot rule out a contribution of anti-TB treatment in the decrease of IFN-γ-releasing cells we observed in patients from group II, several arguments indicate that treatment with TNF antagonists itself largely explains this decline. First, the two patients from group II who did not receive any anti-TB treatment did not differ from the others in terms of evolution of the anti-*Mtb *immune response. Second, IFN-γ release in response to PPD in group I patients (who received no anti-TB treatment) also decreased during treatment with TNF antagonists.

Circulating CD4^+ ^T-lymphocyte numbers remain unaffected in patients treated with TNF antagonists [43,44]. We add functional findings to these previous studies. The preservation of proliferative responses we observed is consistent with the preservation of circulating CD4^+ ^T-lymphocyte numbers. However, the decline of immediate IFN-γ release shows that treatment with TNF antagonists does not affect all aspects of the anti-mycobacterial immune response to the same extent. Memory T lymphocytes have been subdivided into effector/memory (T_EM_) and central/memory (T_CM_) subpopulations. Immediate release of cytokines is typical of T_EM_, which proliferate poorly in response to antigens. In contrast, proliferation is a hallmark of T_CM _(reviewed in [40]). This suggests that TNF antagonists affect anti-mycobacterial CD4^+ ^T_EM _while sparing T_CM_. Expression of chemokine receptors also distinguishes T_EM _from T_CM _(CCR7 and CD62L) [45]. However, lack of defined combinations of *Mtb *peptides and HLA (human lymphocyte antigen) class II tetramers makes difficult the analysis of chemokine receptors on *Mtb*-specific CD4^+ ^T lymphocytes.

The impaired IFN-γ release in patients treated with TNF antagonists indicates that ELISPOT assays should not be used to diagnose previous or latent TB in patients on treatment with TNF antagonists, whereas proliferative assays appear more reliable. Recently approved commercial assays for the *in vitro *detection of anti-TB immunity are based on IFN-γ release and thus, if they are used, should be performed before beginning anti-TNF. Interestingly, Ifx and Eta treatments decreased to the same extent the number of IFN-γ-releasing cells in response to mycobacterial antigens. This possibly contributes to the increased risk of TB infection in patients treated with either Ifx or Eta.

*Ex vivo *studies of anti-mycobacterial immune responses in patients treated with TNF antagonists are useful for evaluating the effect of these agents on circulating resting memory T lymphocytes, but they provide no clue for understanding their effect on lymphocytes recently activated by *Mtb *antigens. To address this issue, we determined whether the *in vitro *addition of TNF antagonists influenced anti-mycobacterial immune response. The results markedly differed from those evaluating the *in vivo *effect of TNF antagonists. *In vitro *addition of TNF antagonists resulted in a decreased proliferation in response to mycobacterial antigens. It also prevented the upregulation of mTNF expression by CD4^+ ^T lymphocytes, another marker of activation induced by mycobacterial antigens. This shows that TNF antagonists either prevent activation of anti-mycobacterial memory T lymphocytes or directly affect activated cells. An effect of TNF antagonists on antigen-presenting cells could account for the first hypothesis. Both Ifx and Eta may induce apoptosis of monocytes/macrophages [46], which are involved in antigen presentation to T lymphocytes and IL-12-mediated activation of these cells. Elimination of monocytes/macrophages may participate in the inhibition of anti-mycobacterial responses. Although we cannot rule out this hypothesis, two findings supported a direct action of TNF antagonists on activated anti-mycobacterial T lymphocytes. First, delaying the addition of the TNF antagonist up to 24 to 48 hours before the end of the culture was sufficient to decrease both proliferation and the fraction of mTNF-expressing cells. Second, Ifx induced a rapid (within 4 hours) and active disappearance of mTNF from the surface of CD4^+ ^T lymphocytes, possibly involving internalization or shedding of mTNF. Regardless of the mechanism(s) involved, a decreased mTNF expression by activated anti-mycobacterial CD4^+ ^T lymphocytes may significantly alter their helper function, considering the role of mTNF on anti-mycobacterial immune responses [19]. In cultures performed with Ifx, apoptosis of activated CD4^+ ^T lymphocytes, expressing mTNF, could be an additional mechanism of inhibition of anti-mycobacterial immune response. This apoptosis could occur through a direct effect of Ifx on either mTNF-expressing cells [47,48] or antibody-dependent cell cytotoxicity. Complement-mediated killing of mTNF-expressing cells is excluded in our experiments, performed in complement-free conditions.

The three TNF antagonists, when added *in vitro*, were not equal in their ability to inhibit the responses induced by mycobacterial antigens. When tested at the 10 μg/ml concentration, inhibition was observed with Ifx and Ada but not with Eta. This observation was not due to an intrinsic inability of Eta to block anti-mycobacterial immune responses, because increasing its concentration up to 40 μg/ml allowed such an inhibition. However, EC_50 _studies indicated that Eta is two to three times less efficient than Ifx and Ada in inhibiting *in vitro *anti-mycobacterial responses. With the exception of CMV antigens, Eta is also less efficient than Ifx and Ada to inhibit the *in vitro *response to recall antigens other than mycobacterial antigens. Although Eta fully neutralises soluble TNF, it is not as efficient as anti-TNF mAbs for binding mTNF-expressed by monocytes/macrophages and Jurkat T lymphocytes [16,47,48]. Because mTNF expression is upregulated on CD4^+ ^T lymphocytes activated by mycobacterial antigens, the anti-TNF antibodies may alter the function of activated anti-*Mtb *T lymphocytes more than Eta. These alterations may occur *in vivo *in patients with TB reactivation given that they were observed *in vitro *at concentrations of TNF antagonists (1–10 μg/ml) corresponding to their therapeutic range [2,38]. Therefore, TB reactivation may stimulate anti-*Mtb *T lymphocytes and expose them to inhibition or deletion by anti-TNF mAbs. This could explain why TB reactivations in patients treated with TNF antagonists are so severe and disseminated, with few granulomas in involved tissues, and why they might be more frequent with Ifx than with Eta.

## Conclusion

This work shows that TNF antagonists have a dual effect on anti-mycobacterial CD4^+ ^T lymphocytes. Their *in vivo *administration results in a decline of subpopulations of anti-mycobacterial memory CD4^+ ^T lymphocytes, especially those rapidly releasing IFN-γ upon challenge with mycobacterial antigens. All TNF antagonists tested appear as efficient to induce this decline. In contrast, anti-TNF mAbs are more efficient than Eta in inhibiting the *in vitro *activation of CD4^+ ^T lymphocytes by mycobacterial antigens, presumably reflecting their higher potency in interacting with mTNF expressed by activated CD4^+ ^T lymphocytes. Such a dual effect may explain the increased incidence of TB in patients treated with TNF antagonists as well as possible differences between TNF antagonists for the incidence and the clinical presentation of TB reactivation.

## Abbreviations

Ada = adalimumab; BCG = Bacille de Calmette Guérin; CD = Crohn's disease; Cd = *Candida*; CFP-10 culture filtrate protein-10; CMV = cytomegalovirus; EC_50 _= median effective concentration; ELISPOT = enzyme-linked immunosorbent spot; ESAT-6 = early secretory antigen target-6; Eta = etanercept; IFN = interferon; Ifx = infliximab; IL = interleukin; IMID = immune-mediated inflammatory disease; mAb = monoclonal antibody; *Mtb *= *Mycobacterium tuberculosis*; mTNF = membrane-bound tumour necrosis factor; PBMC = peripheral blood mononuclear cell; PPD = purified protein derivative (or tuberculin); RA = rheumatoid arthritis; SA = sponlylarthropathy; TB = tuberculosis; TNF = tumour necrosis factor; TNFR = tumour necrosis factor receptor; Toxo = *Toxoplasma gondii*; TT = *Tetanus toxoid*.

## Competing interests

The authors declare that they have no competing interests.

## Authors' contributions

HH, VG, and MVP evaluated the anti-mycobacterial immune responses. XM, DE, and the RATIO (Recherche sur Anti-TNF et Infections Opportunistes) Study Group designed the study. HH, XM, and DE were in charge of the redaction of the manuscript. KW produced the *Mtb *antigens. GB was in charge of statistical studies. All other authors (AMH, ML, XP, MB, FB, JCD, RMF, BD, DS, and MH) were involved in the recruitment and monitoring of patients. HH and XM contributed equally to this work. All authors read and approved the final manuscript.

## Figures and Tables

**Table 1:** Characteristics of patients

	*Group I*	*Group II*	*Group III*	*Group IV*
*n*	37	31	21	24

Age (median, IQR)	43 (31–59)	52 (42–56)	65 (37–72)	60 (50–72)
Gender (male/female)	19/18	13/18	11/10	14/10
IMID* (RA/SA/CD)	13/12/12	16/13/2	0	0
TNF antagonists*				
RA (Ifx/Ada/Eta)	11 (0/1/10)	12 (3/2/7)	0	0
SA (Ifx/Ada/Eta)	9 (2/1/6)	13 (8/1/4)		
CD (Ifx/Ada/Eta)	11 (11/0/0)	0 (0/0/0)		
Previous anti-TB treatment	0	2	0	20
Associated immunosuppressors (MTX/AZA/CT)	17/6/18	14/1/11	0	0
TST (≥10 mm)	0/37	31/31	0/21	24/24

*A few patients were studied at inclusion but not at week 14. Ada, adalimumab; AZA, azathioprine; CD, Crohn's disease; CT, corticosteroids (≤10 mg/day); Eta, etanercept; Ifx, infliximab; IMID, immune-mediated inflammatory disease; IQR, interquartile range; MTX, methotrexate; RA, rheumatoid arthritis; SA, spondylarthropathy; TB, tuberculosis; TNF, tumour necrosis factor; TST, tuberculin skin test.
